# High mesh density flow diversion of upper basilar artery aneurysms: Experience in 23 cases

**DOI:** 10.1177/15910199251385633

**Published:** 2025-10-17

**Authors:** Jessica K Campos, Jonathan C Collard de Beaufort, Fahad J Laghari, Kimberlee A Van Orden, Melinda C Arthur, David A Zarrin, Benjamen M Meyer, Gizal A Amin, Narlin B Beaty, Matthew T Bender, Shuichi Suzuki, Geoffrey P Colby, Alexander L Coon

**Affiliations:** 1 Department of Neurological Surgery, 8788 University of California Irvine, Orange, CA, USA; 2 12302 SUNY Upstate Medical School, Syracuse, NY, USA; 3 21944 Carondelet Neurological Institute, St Joseph's Hospital, Tucson, AZ, USA; 4 12222 University of California Los Angeles, David Geffen School of Medicine, Los Angeles, CA, USA; 5 12216 University of Arizona, College of Medicine, Tucson, AZ, USA; 6 22163 Florida State University, Tallahassee Memorial Hospital, Tallahassee, FL, USA; 7 6927 University of Rochester, Department of Neurosurgery, Rochester, NY, USA

**Keywords:** Basilar, flow diversion, posterior circulation, mesh density

## Abstract

**Background:**

Flow diversion has proven feasible for treating posterior circulation aneurysms. These studies, however, are limited to the utilization of low-mesh density devices. The high-mesh density and mesh geometry of the Surpass Evolve and Streamline flow diverters theoretically allow increased flow diversion while preserving perforators, a critical consideration in posterior circulation flow diversion. We investigate this phenomenon in the largest known series utilizing solely high-mesh density devices for basilar aneurysms.

**Methods:**

A prospectively maintained, IRB-approved database of the senior authors was retrospectively reviewed for flow diversion cases involving the upper basilar artery and utilizing Surpass Evolve or Streamline. Technical success was defined as the successful implantation of the device without intraprocedural device removal.

**Results:**

Over a 52-month study period, 23 cases were identified utilizing Surpass Evolve (21 devices) and Surpass Streamline (2 devices) treating aneurysms located along the basilar apex (12 cases, 52%) and superior cerebellar artery (11 cases, 48%). Technical success was achieved in all cases (23 cases, 100%). Patients were placed on antiplatelet therapy consisting of aspirin (22 cases, 95.7%) and ticagrelor (20 cases, 87%) or prasugrel (4 cases, 17.4%). There was one mortality within the 30-day postoperative window due to complications of the presenting high-grade SAH.

**Conclusion:**

The treatment of upper basilar and basilar apex aneurysms with high mesh density devices can be performed with acceptable safety standards. Further studies are needed to confirm the safety profile, occlusion rates, and applicability of these findings to lower mesh-density 48-wire flow-diverter implants at the basilar apex.

## Introduction

Flow diversion has revolutionized the field of endovascular neurosurgery by redirecting blood flow away from the aneurysm sac and providing durable and curable aneurysm occlusion. This has been demonstrated numerous times to be a safe and effective method of aneurysm treatment across a variety of anatomical variations, including side-wall aneurysms. There is a paucity of flow diversion reports on bifurcating aneurysms (aneurysms located at the branch point off the parent vessel) and perforator-rich environments such as the anterior communicating artery and upper basilar artery.

The safety of flow diversion in the posterior circulation, and more specifically in the proximal basilar artery, with low-mesh density flow diverters has been well documented across multiple studies.^1–4^ These reports used the Silk (Balt Extrusion, Montmorency, France), Derivo (Acandis GmbH & Co. KG, Pforzheim, Germany), Tubridge Embolization Device (MicroPort NeuroTech, Shanghai, China), Pipeline Embolization Device (PED; Medtronic Neurovascular, Irvine, California, USA), or Flow Redirection Endoluminal Device (FRED; MicroVention, Aliso Viejo, California, USA) which are either 48- or 64-braid. The safety and efficacy of high-mesh density flow diverters such as Surpass Evolve or Surpass Streamline (Stryker Neurovascular, Freemont, California, USA) is well established in the literature.^5,6^ However, other than a single case report, no large studies report on flow diversion in the basilar apex complex using high-mesh density flow diverters.^7^

We present, to the best of our knowledge, the largest case series describing the safety profile of high-mesh density flow diverters deployed in the basilar circulation[Table table1-15910199251385633][Table table2-15910199251385633][Table table3-15910199251385633].

**Table 1. table1-15910199251385633:** Case summary.

	Number/range	Percent/SE
*Demographics*		
Total cases	23	
Average age (years)	67.7 (41–90)	± 2.6
Female sex	18	78.3%
Presenting symptoms		
Routine	12	52.2%
Headache	3	13%
Subarachnoid hemorrhage	2	8.7%
Trauma	1	4.3%
Other	5	21.7%
*Aneurysm details*		
Average size	4.6	± 0.7
Aneurysm types		
Saccular	17	73.9%
Ruptured	2	8.7%
Complex	2	8.7%
Irregular	1	4.4%
Blister	1	4.4%
Aneurysm locations		
Apex	12	52.2%
SCA	11	47.8%

**Table 2. table2-15910199251385633:** Procedural details.

	Number/range	Percent/SE
*Case characteristics*		
Fluoroscopy time (minutes)	26.0	± 3.7
Contrast amount (mL)	44.7	± 8.0
Radiation exposure (mGy)	915	± 101
*Access and catheters*		
Access artery		
Femoral	19	82.6%
Radial	4	17.4%
Catheter system		
Triaxial	23	100%
Biaxial	0	0%
DAPT		
Aspirin^a^	22	95.7%
Ticagrelor^b^	20	87.0%
Prasugrel^c^	4	17.4%
Long guide position		
V2	12	52.2%
V3	5	21.7%
V4	4	17.4%
Proximal vertebral	2	8%
Distal intracranial position		
V4	13	56.5%
Basilar	7	30.4%
V3	1	4.3%
V2	1	4.3%
PCA	1	4.3%
*Devices used*		
FDS average width		
Surpass evolve (*n* = 21)	4.3	0.07
Surpass streamline (*n* = 2)	3.0	0.00
VRD average width		
Atlas (*n* = 1)	3	
Procedural outcomes		
Technical success	23	100%
Stent migration	0	0%

DAPT: dual antiplatelet therapy; FDS: flow diverting stent; VRD: vascular reconstruction device; PCA: posterior cerebral artery.

a Patients were either on aspirin 81 or 325 mg.

b Patients were either on ticagrelor 60 or 90 mg.

c Patients were on prasugrel 10 mg.

**Table 3. table3-15910199251385633:** Patient outcomes.

	Number/range	Percent/SE
*Aneurysm occlusion*		
Case with follow-up	19	82.6%
Follow-up time (months)	12.9	± 2.1
Raymond grade		
1	13	68%
2	4	21%
3	2	11%
*Complications*		
Mortality	1	4.35%
Rupture/perf intraoperatively	0	0%
In-situ thrombosis	0	0%
Transient deficit	0	0%
CN palsy	0	0%
Iatrogenic dissection	0	0%

## Methods

A prospectively maintained, IRB-approved database of senior authors’ cases was retrospectively reviewed for flow diversion cases involving the basilar apex complex artery and utilizing Surpass Evolve or Streamline. Aneurysm measurements were obtained from 2D catheter angiography images, with aneurysm size defined as the maximal dimension of the sac. Proper action was taken to achieve appropriate anonymity for this manuscript. All averages are reported with their standard error.

## Results

A total of 23 cases of Surpass flow diversion at the basilar apex complex were identified (Table 1). There were 18 females (78%) and the average age was 67.7 ± 2.6 years. Aneurysms were either discovered incidentally (13 cases, 57%), during headache workup (3 cases, 13%), after subarachnoid hemorrhage (2 cases, 9%), after other neurological symptoms (4 cases, 17%), or after trauma (1 case, 4%). Aneurysm maximum diameter averaged 4.6 ± 0.7 mm, with 2 aneurysms being ruptured (9%). Twelve cases (52%) involved a bifurcation aneurysm.

A total of 23 Surpass flow diversion devices were deployed (21 Evolve, 2 Streamline), with an average diameter and length of 4.2 ± 0.1 and 17.5 ± 0.7, respectively. In 3 cases (13%), adjunct coiling was used. No in situ thrombosis was noted in any case. In total, 4 (17%) of cases were performed radially. There were 4 cases with concern for device stenosis and poor apposition which was treated with off-label use of the Comaneci device (Rapid Medical, Yokneam, Israel) for augmenting vessel wall apposition.^6^

Patients were placed on dual antiplatelet therapy periprocedurally, with aspirin and ticagrelor in 18 cases (78%), aspirin and prasugrel in 3 cases (13%). One case (4%) had triple antiplatelet therapy and another case (4%) had mono antiplatelet therapy.

Follow-up was available in 19 cases (83%), with an average time of 12.9 ± 2.1 months, demonstrating Raymond grade 1 occlusion in 13 cases (68%), Raymond grade 2 in 4 (21%), and Raymond grade 3 in 2 (11%). Follow-up was not available in 4 cases (17%), one of which (1 case, 4%) was due to mortality. On follow-up, flow limiting in-stent stenosis was absent in all cases (100%). There were no ischemic or hemorrhagic complications at follow-up.

## Discussion

Flow diversion in bifurcating aneurysms has been well described in the anterior circulation, particularly in middle cerebral artery bifurcations and anterior communicating artery aneurysms. Basilar apex bifurcation aneurysms, by contrast, present unique anatomical and technical challenges due to a perforator-rich environment and the potential need to cover major branch vessels, such as the posterior cerebral arteries (PCAs).^8^ Our study focused exclusively on the use of Surpass Evolve and Streamline flow-diverting devices, which have higher mesh density compared to other flow diverters (Table 2). Our study supports that high-mesh density stents offer high aneurysm occlusion rates while maintaining patency of critical perforators.

The basilar apex is inherently a bifurcating zone (with the PCAs taking off), and aneurysms in this region have traditionally been endovascularly addressed via stent-assisted coiling or Y-stent coiling techniques. However, such approaches have been associated with a comparatively higher rate of morbidity. A multicenter study by Fargen et al.^9^ on Y-stent coiling of basilar apex aneurysms reported 20% procedure-related complications. This has been corroborated in prior smaller and single-center series.^10–12^

Early reports of flow diversion in the posterior circulation predominantly used lower mesh density devices such as the PED. Bender et al.^13^ published a series on flow diversion of posterior circulation aneurysms, including seven basilar apex aneurysms, in which one patient (14%) experienced a perforator ischemic complication. Notably, this occurred in a patient who was on clopidogrel and not high-potency ticagrelor. Although that paper established feasibility, the complication rate highlights the delicate nature of posterior circulation aneurysm interventions, particularly in the basilar apex. In contrast to the studies referenced above, we observed no device-related ischemic events in our larger and focused series while achieving high aneurysm occlusion rates. We speculate that this finding may relate to the higher mesh density of the Surpass devices, as well as our progressive practice of more potent antiplatelet regimens with prasugrel and ticagrelor.

Flow-diverting stents function as endoluminal scaffolds, redirecting blood flow away from the aneurysm sac while preserving flow through critical perforators and branch vessels based on hemodynamic demand. Specifically, two major parameters including porosity and mesh density must be carefully balanced to achieve effective flow reduction into the aneurysm sac without compromising adjacent vessels.^14^ High-mesh density flow diverters such as the Surpass device, with its numerous small pores, theoretically has a higher likelihood of maintaining perfusion in perforator arteries driven by local hemodynamic requirements, while still diverting a substantial portion of flow away from the aneurysm sac. We believe that this balance may be the key to preserving critical branches and minimizing ischemic complications such as in this series.

Equally important in the treatment of basilar aneurysms is antiplatelet therapy.^15^ It is important to emphasize that, in this series, dual antiplatelet therapy with aspirin plus ticagrelor or prasugrel was our standard regimen. Unlike clopidogrel, prasugrel and ticagrelor demonstrate near-zero resistance rates and greater intrinsic potency by P2Y12 testing. Our preferred agent is ticagrelor 90 mg twice daily; however, we use prasugrel when patients experience side effects, when adherence is a concern due to twice-daily dosing, or when affordability is an issue. We believe this regimen mitigates the risk of in-stent thrombosis, particularly in perforator-rich regions such as the basilar apex.

The occlusion rates we observed (89% Raymond 1 and 2 occlusion at a mean follow-up of 12.9 months) are encouraging, particularly given that no patient in our series required subsequent treatment (Table 3). Basilar apex aneurysms have long proven difficult to cure with more conventional coiling and stent-coiling techniques.^16,17^ The ability of high-mesh density implants to reconstruct the parent artery and redirect blood flow away from the aneurysm sac appears to be a key factor in achieving long-term curative occlusion.^18^ Additionally, the configuration of collateral circulation, in particular, the presence of a posterior communicating artery ipsilateral to the covered/jailed PCA, can effectively reduce the hemodynamic demand through the jailed PCA. This essentially converts a bifurcation aneurysm into a sidewall aneurysm for the purpose of flow diversion. When this anatomical configuration exists, flow diversion in the basilar apex might be argued to be the superior strategy.

These findings highlight the potential advantages of high-mesh density flow diverting devices and robust antiplatelet therapy for basilar apex aneurysms and potentially represent a strong combined treatment strategy. Future studies with larger cohorts and longer follow-up will be essential to confirm these results and further optimize patient selection and treatment paradigms.

**Figure 1.**
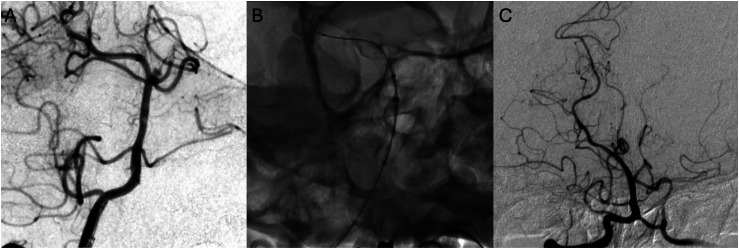
Case 1 (A–C): A blister basilar apex aneurysm treated with Surpass Streamline embolization device. A two-month follow-up shows Raymond grade 1 occlusion of the aneurysm and absence of anterograde flow in the left posterior cerebral artery (PCA), converting this bifurcation aneurysm into a sidewall aneurysm.

**Figure 2.**
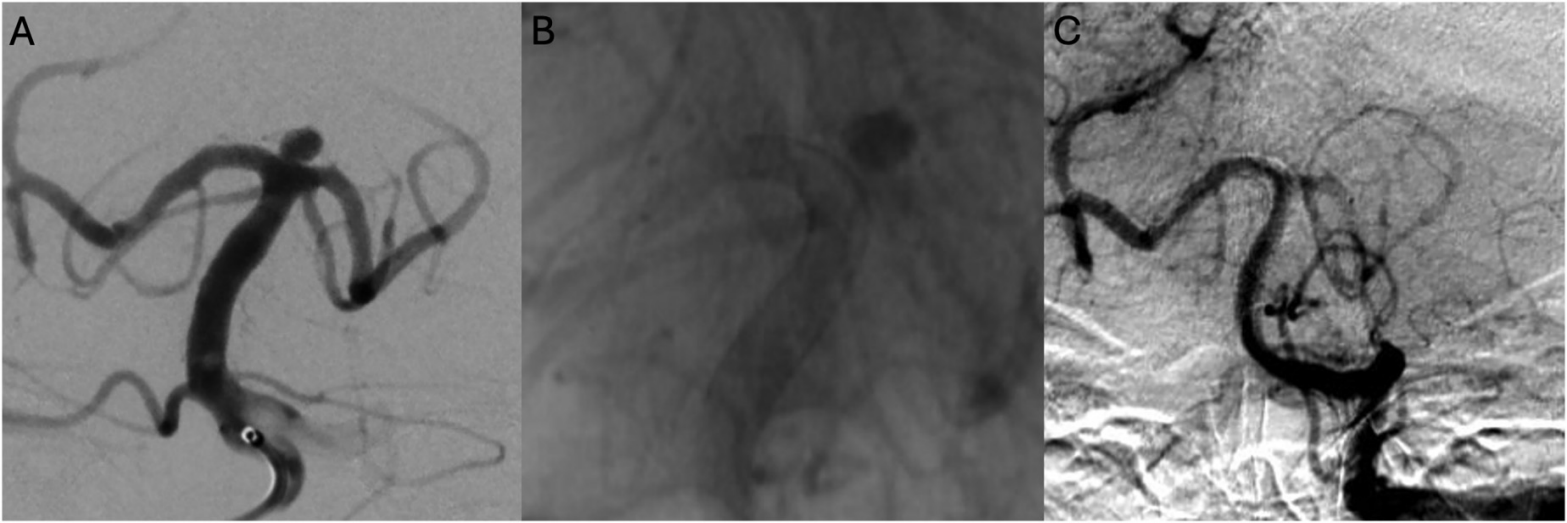
Case 2 (A–C): A 4 mm wide-necked basilar apex aneurysm treated with Surpass Evolve embolization device. A six-month follow-up shows Raymond grade 1 occlusion of the aneurysm.

Case illustration in this series (Figure 1) demonstrates Raymond grade 1 occlusion on the last follow-up of a basilar apex aneurysm. The aneurysm was treated solely utilizing a Surpass Evolve flow diverter, deployed from the PCA to the basilar artery (Figure 1B). Periprocedurally, intra-arterial tirofiban was administered prophylactically to prevent thrombus formation and intra-arterial verapamil for vasospasm prophylaxis. Prolonged intra-arterial verapamil infusion, and intra-arterial tirofiban administration after stent placement were standard in our series. This patient achieved Raymond Grade 1 on 6-month follow-up. Other than a 4% mortality rate in a patient who presented with a subarachnoid hemorrahage prior to FDS treatment and succumbed to SAH-related complications, no complications occurred in the series. Figure 2 demonstrates a 4-mm wide-necked basilar apex aneurysm treated with Surpass Evolve embolization device with Raymond grade 1 occlusion at six-month follow-up.

## Limitations

While this study offers valuable insights into the treatment of upper basilar artery aneurysms with flow diversion, it is limited by its retrospective design, relatively small cohort, and absence of a comparator group for outcome benchmarking. Although a 12.9-month follow-up period is often sufficient for most flow diversion series, these aneurysms can be especially recalcitrant and may warrant longer-term follow-up to fully assess the true durability of this strategy. Furthermore, this technique may require prolonged antiplatelet therapy, and the long-term effects of such a regimen cannot be fully evaluated in a shorter-term analysis such as this.

## Conclusion

Our experience suggests both safety and efficacy of treatment of basilar apex and upper basilar artery aneurysms utilizing high mesh density flow diverters. Flow diversion using high-potency DAPT resulted in effective treatment with no evidence of perforator infarction in any patient. Although additional and larger multi-center studies are necessary to confirm these results, our preliminary findings suggest that flow diversion with a robust antiplatelet regimen is a viable treatment option for carefully selected posterior circulation aneurysms.
